# Vertical Distribution Characteristics and Ecological Risk Assessment of Mercury and Arsenic in Ice, Water, and Sediment at a Cold-Arid Lake

**DOI:** 10.3390/toxics12080540

**Published:** 2024-07-26

**Authors:** Zhimou Cui, Shengnan Zhao, Xiaohong Shi, Junping Lu, Yu Liu, Yinghui Liu, Yunxi Zhao

**Affiliations:** 1 Water Conservancy and Civil Engineering College, Inner Mongolia Agricultural University, Hohhot 010018, China; 2 Water Resources Protection and Utilization Key Laboratory, Inner Mongolia Agricultural University, Hohhot 010018, China; 3 State Gauge and Research Station of Wetland Ecosystem, Wuliangsuhai Lake, Bayan Nur 014404, China

**Keywords:** single-factor pollution index, geo-accumulation index, non-carcinogenic risk, carcinogenic risk, Monte Carlo simulation, sensitivity analysis

## Abstract

Mercury and arsenic are two highly toxic pollutants, and many researchers have explored the effects of the two substances on the environment. However, the research content of toxic substances in frozen periods is relatively small. To explore the spatial and vertical distribution of mercury and arsenic in the ice, water, and sediments of Wuliangsuhai Lake under ice conditions, and to assess the harm degree of the two toxic substances to human beings. We collected the ice, water, and sediments of the lake in December 2020, and tested the contents of Hg and As. The single-factor pollution index method, the local cumulative index method, and the ecological risk coding method were used to assess the pollution status in these three environmental media, and the Monte Carlo simulation combined with the quantitative model recommended by USEPA was used to assess the population health risk. The results showed that (1) The average single-factor pollution values of Hg and As in water were 0.367 and 0.114, both pollutants were at clean levels during the frozen period. (2) The mean Igeo values of Hg and As were 0.657 and −0.948. The bioavailability of Hg in the sediments of Wuliangsuhai Lake during the frozen period was high, and its average value was 7.8%, which belonged to the low-risk grade. The bioavailability of As ranged from 0.2% to 3.7%, with an average value of 1.3%. (3) Monte Carlo simulation results indicate acceptable levels of health risks in both water and ice. This study preliminarily investigated the distribution characteristics of toxic substances and their potential effects on human health in lakes in cold and arid regions during the frozen period. It not only clarified the pollution characteristics of lakes in cold and arid regions during the frozen period, but also provided beneficial supplements for the ecological protection of lake basins. This study lays a foundation for further environmental science research in the region in the future.

## 1. Introduction

Mercury and arsenic are naturally occurring elements that are widely distributed in the environment [1]. These two elements are highly toxic [2]. Mercury is a global pollutant that possesses unique physicochemical properties, such as volatility, capacity for long-range transport, persistence, and bioaccumulation [3]. Mercury can enter water through various pathways, including atmospheric dry and wet deposition [4,5] and surface runoff [6]. In the presence of microorganisms, it can be transformed into a highly toxic compound called methyl mercury (MeHg) [7], which poses a significant risk to human health through the food chain. The disturbance of mercury-bearing sediment [4,8] can result in its release of this element into the water column, leading to water pollution. Microscopic transport processes also contribute to the release of Hg from sediment [9]. Arsenic is highly toxic and carcinogenic [10] and tends to accumulate in aquatic plants, algae, and fish, posing a potential effect to ecosystems [11]. Arsenic can persist in water for up to 100 years [12]. Acute or chronic exposure to As can cause severe damage to the skin, cardiovascular system, and the cerebrovascular system in humans [13].

Wuliangsuhai Lake is sometimes called Ulansuhai Lake, located in the Yellow River basin. It is the largest freshwater lake in China and one of eight freshwater lakes in the country. It is also the largest wetland in the world at its latitude [14]. Additionally, Wuliangsuhai Lake is a highly representative cold-zone lake [15], possessing a significant ecological service and river research value. Lakes in the cold regions of China are characterized by long frozen periods and relatively thick ice layers. During winter, the presence of ice significantly alters the physical and chemical properties of the water. The chemical cycle of pollutants in the environmental medium of lake ice exhibits distinct characteristics, directly impacting the water’s ecological environment during the frozen period. As the freezing process involves the transformation of water from liquid to solid, the concentration of unfrozen water pollutants increases [15]. Wuliangsuhai Lake is an important farmland drainage site in the arid region of northwest China. The use of pesticides in agricultural production activities leads to the increase in Hg and As contents in the upstream water body. Previous studies have shown that Hg and As in the water environment of Wuliangsuhai Lake are at a high level [16]. Consequently, this exacerbates the deterioration of the water environment during the period of ice cover. Therefore, conducting research on the migration and transformation patterns of pollutants during the frozen period of lakes in cold regions is of utmost importance.

Most previous studies have primarily focused on the concentration and morphological characteristics of heavy metals in unfrozen water or sediments [17,18]. However, there is a lack of statistical data regarding the presence of heavy metal elements in different media during the frozen period, as well as the mechanisms governing the behavior of heavy metals under changing environmental conditions, particularly during the process of ice cover and thawing. Therefore, this study aims to investigate the content and distribution of Hg and As in various media within the frozen Wuliangsuhai Lake including ice, water, and sediment, and use Monte Carlo simulation to evaluate the risks to humans caused by the two toxic substances. By examining the geochemical characteristics, biological effectiveness, and environmental impact of Hg and As during the freezing process, this research will contribute significantly to the understanding of frozen lake pollution control in cold regions and provide a scientific basis for effective management strategies.

## 2. Materials and Methods

### 2.1. Study Area

Wuliangsuhai Lake is situated in Urat Front Banner, Bayannur City, Inner Mongolia Autonomous Region (40°36′~41°03′ N, 108°43′~108°57′ E). In some research, Ulansuhai Lake also refers to this area [16]. It is one of eight freshwater lakes in China and is considered a typical shallow lake in a cold and arid region. It decreases in width from east to west and from north to south. As of 2020, the total area of the lake was 341 km^2^, with a water volume of 400–500 million m^3^. Located in the northern cold and arid region of China, Wuliangsuhai Lake experiences a typical mid-temperate continental monsoon climate characterized by dryness, low rainfall, high evaporation rates, and distinct seasonal variations in temperature and precipitation. The lake enters its frozen period every November and remains frozen for approximately five months until the end of March or early April [19]. During this period, the ice thickness ranges from 0.4 to 0.6 m, and the depth of water under ice is 0.8 m–1.5 m.

### 2.2. Sample Collection and Evaluation

**Figure 1:**
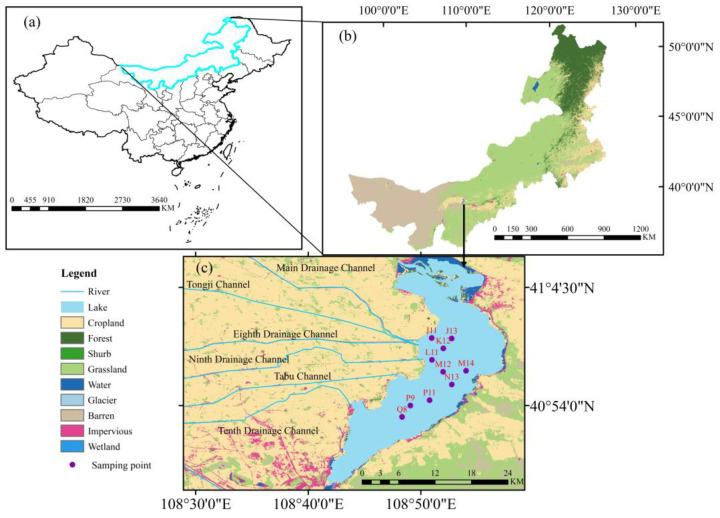
Location of the study area and sampling points. (**a**). China’s border, (**b**) Inner Mongolia Autonomous Region, (**c**) Study area and sampling points. For name of sampling points, letters indicate the location of the waterway, and the number is only used to distinguish different sampling points.

During the routine monitoring of the frozen period, the area was divided using a grid based on the actual situation of the lake area. A GPS system was utilized to determine the locations at which samples of lake ice, ice water, and sediment were collected. Ten sampling points were chosen based on taking into consideration factors such as water depth, ice thickness, presence of aquatic plants, and relatively high levels of heavy metal pollution in the water (Figure 1). To ensure experimental rigor, sampling was conducted in December 2020, when the water had completely frozen and the physical and chemical properties of the water and sediment had stabilized. The GPS system was used to record the locations of the ice samples and ice water samples, and a YSI water quality analyzer (YSI Pro 1020, YSI Company, Cleveland, OH, USA) was employed on site to measure the physical and chemical properties of the water. The specific operation is as follows: after opening the ice, insert the YSI (YSI Pro 1020, YSI Company, Cleveland, OH, USA) device from the broken ice cave into the water below the ice, and use different sensing probes on the YSI (YSI Pro 1020, YSI Company, Cleveland, OH, USA) device to measure the conventional environmental indicators in the water, including pH, temperature, and salinity. Finally, sediment samples were collected and sealed in packages for transportation to the laboratory for testing. The specific steps for collecting different samples were as follows:

The ice sample collection process was as follows: At every sampling point, an ice collector was used to remove a 50 cm × 50 cm ice block. A chainsaw was used to vertically cut the ice, and the ice was removed in the field. The ice was then transported to the laboratory. The ice was compressed from top to bottom and was cut at intervals of 5 cm. Each piece was placed in a plastic sealing bag, awaiting the melting test; 100 ice samples were collected in total.

The water sample collection was as follows: After removing the ice, a custom-made layered water intake device was slowly placed into the water, allowing the lake water to gradually fill up from the bottom of the water intake and fill each layer. The height of each layer of the water intake unit was 5 cm. The process continued until the bottom layer of the water intake reached the muddy water interface. The tensile thread steel bar was lifted upward, and the bolts on the upper plate of the water intake device were tightened with a wrench. This ensured that the lower end of the water intake container for each layer could close the water intake for that layer. Finally, the entire water sample and the water intake were collected. The lateral spring clips of each layer were opened, and water samples from each layer were transferred into polyethylene plastic bottles according to the layer’s serial number (Figure 1). In total, 100 water samples were collected, sealed, and transported to the laboratory for testing.

The sediment sample collection was as follows: Sediment samples were collected after ice and water samples were collected. Using the gravity columnar sampler (UWITEC, Innsbruck, Austria), approximately 30 cm of sediment column samples were collected and layered in 5 cm layers on site. They were then quickly packed into polyethylene plastic bags and stored for testing. The length of the column samples ranged from 10 to 30 cm. The stratification of sediment samples was as follows: The J13 sample was divided into five layers, while the N13 and Q8 samples were divided into four layers. The K12, L11, M12, M14, and P9 samples were divided into three layers. The J11 and P11 samples were divided into two layers. Once the sediment samples were collected, they were carefully placed in self-sealed bags and transported to the laboratory for further analysis. The selection of sampling points followed safety and scientific principles. Each sampling point was a long-term observation sampling point, close to waterways or reed areas, and was affected by certain non-natural factors. A total of 32 sediment samples were collected.

When collecting water samples, they were subjected to various tests using YSI equipment (YSI Pro 1020, YSI Company, Yellow Springs, OH, USA). These tests included measurements of pH, REDOX potential (ORP), conductivity (EC), turbidity (Turb), and total dissolved solids (TDS). In the laboratory, the sediment samples were analyzed for total nitrogen (TN), total organic carbon (TOC), and salinity (SAL). The equipment underwent sensitivity testing and the detection limit met the requirements, with an error of less than 1% in the standard solution. When using the equipment, the readings must stabilize before recording, and 3 tests on each sample were carried on site to obtain the average value.

#### 2.2.1. Detection of Mercury and Arsenic

In the laboratory, the water and ice samples for determination of mercury were treated with hydrochloric acid at a concentration of 5 mL per liter, while the water and ice samples for determination of arsenic were treated with hydrochloric acid at a concentration of 2 mL per liter. The ice and water samples that had undergone pretreatment were tested within a period of 14 days. The concentrations of total mercury (THg) in the water and ice samples were determined following Method 1631 of the United States Environmental Protection Agency (USEPA) [20]. According to this method, 0.125 mL of 0.2 N BrCl was added to 25 mL of the lake water sample and allowed to react for 12 h. Subsequently, 0.0625 mL of 30% *w/v* NH_2_OH·HCl was added to remove any residual BrCl. Then, 0.125 mL of 20% *w/v* SnCl_2_ was added to convert all Hg^2+^ to Hg^0^. The generated Hg^0^ was then detected using a MERX automated modular Hg system (Brooks Rand Labs, Seattle, WA, USA). The concentration of As after pretreatment was determined using an atomic fluorescence spectrometer (Kylin S18 Jitian, Beijing, China). Before the measurement, the process involves the following: take 50.0 mL of the water sample and transfer it to a 150 mL conical flask. Add 5 mL of HNO_3_—HClO_4_ mixed acid (*v*/*v* = 1:1) and heat it on an electric heating plate until white smoke appears, then cool it down. Add 5 mL of HCl solution again, heat until all yellow brown smoke is emitted, cool down, transfer to a 50 mL volumetric flask, dilute with water to volume, mix well, and wait for measurement. The detection limits for Hg and As are 0.2 ng/L and 0.3 μg/L.

After the sediment samples were transported to the laboratory, the impurities, such as plant residues and large particles, were eliminated. The samples were passed through a 100-mesh sieve, freeze-dried, and stored in light-sealed bags for the determination of the total amount of As and Hg, as well as the content of each form.

To measure the total Hg and total As concentrations, 0.2 g of the sediment sample was digested with a mixture of HCl and HNO_3_ in a 3:1 (*v*/*v*) ratio using a microwave digestion system (Multiwave PRO, Anton Paar, Graz, Austria). The setting conditions are as follows: heat the temperature to 120 °C within 6 min, keep it at 120 °C for 6 min, then heat it up again to 180 °C and keep it for 15 min. After digestion, the supernatant was filtered through a 0.45 μm filter, cooled to room temperature, and diluted with deionized water to a final volume of 50 mL. The analysis was then conducted using an atomic fluorescence spectrometer (Kylin S18 Jitian, Beijing, China) [21]. The detection limits for Hg and As are 0.002 mg/kg and 0.01 mg/kg.

The different forms of As and Hg were determined based on the BCR metal morphology classification method [22]. The forms of As and Hg were categorized as follows: acid extractable (F1), iron/manganese oxide bound (F2), organic and sulfide bound (F3), and residue (F4). Among these forms, F1, F2, and F3 are typically considered convertible states, which can be released into the overlying water under conditions of weak acetic acid, reduction, and oxidation, thereby posing ecological risks to the water. In contrast, the F4 form is relatively stable.

#### 2.2.2. Quality Assurance and Quality Control

Samples were conducted in strict accordance with quality assurance/quality control (QA/QC) measures. The containers used in the experiment were soaked in an acid tank for 24 h and cleaned. During the experiment, blank samples and parallel samples were set up, and all samples were subjected to 3 parallel samples. The result was taken as the average of the 3 test analyses, with an error range of less than 10%. At the same time, blank analysis was performed every 10 samples. For better control of the quality of the analysis, the standard soil (GBW07451, Langfang, China) [23] and standard liquid (GBWE084161 and GBWE084159, Beijing, China) [24,25] were added, the recoveries for contents were between 92.2% and 103.2%. Test results showed that the blank values were 1~4% of the measured sample value.

### 2.3. Methods and Analysis

#### 2.3.1. Sediment Quality Guidelines (SQGs)

As standards for lake sediments have not yet been established in China, this study adopted the internationally recognized sediment quality guidelines (SQGs) to assess the potential effect of heavy metals in sediments from lakes, rivers, and other water. The SQGs employ the Threshold Effect Level (TEL) and Probable Effect Level (PEL) to represent critical and possible concentrations for adverse effects, respectively. If the heavy metal content is below the TEL, there are almost no biotoxic effects. If the content is between the TEL and PEL, biotoxic effects occur occasionally. If the content exceeds the PEL, there is a high likelihood of biotoxic effects [26].

#### 2.3.2. Single-Factor Pollution Index

The level of As and Hg pollution in water was evaluated using the single-factor pollution index. The method uses the ratio between the actual measured content of specific heavy metals in different environmental media and the established evaluation standards to quantify the degree of environmental pollution caused by specific heavy metals in the environment. It is one of the most widely employed assessment methods [27,28]. The formula used for calculation is as follows:

(1) $$P_{i}=\frac{C_{i}}{S_{i}}$$

In this study, the contamination index (*P_i_*) was used to assess the level of pollution. The measured value of the element (*C_i_*) was compared to the standard value (*S_i_*), which was determined based on the basic control item limit in the standard for irrigation water quality (GB5084-2021) [29]. The standard values for Hg and As were 1 μg/L and 50 μg/L, respectively. The evaluation criteria for the single-factor pollution index are as follows: clean (*P_i_* < 1), slight pollution (1 ≤ *P_i_* < 2), moderate pollution (2 ≤ *P_i_* < 3), and severe pollution (*P_i_* ≥ 3) in the water.

#### 2.3.3. Geo-Accumulation Index (*I_geo_*)

This method considers the geochemical background value of the environment, natural diagenesis, and human activities. Consequently, it is extensively employed in the assessment of heavy metal pollution levels in soil and sediment [30,31]. The calculation formula is as follows:

(2) $$I_{geo}=\log_{2}\left[\frac{C_{n}}{KB_{n}}\right]$$

where *C_n_* represents the measured content of heavy metal element n in mg/kg, *B_n_* represents the environmental background value of the measured heavy metals, and *K* is the fixed value for the natural fluctuation of heavy metal content (*K* = 1.5). The reference value for *B_n_* is selected as the background value of drainage sediment in Inner Mongolia Autonomous Region, the background value of Hg is 0.025 mg/kg, and the background value of As is 9.68 mg/kg [16].

The degree of heavy metal pollution corresponding to the *I_geo_* value is divided into seven levels:

no pollution (*I_geo_* ≤ 0), mild pollution (0 ≤ *I_geo_* < 1), partial moderate pollution (1 ≤ *I_geo_* < 2), moderate pollution (2 ≤ *I_geo_* < 3), heavy pollution (3 ≤ *I_geo_* < 4), severe pollution (4 ≤ *I_geo_* < 5), and extremely severe pollution (*I_geo_* ≥ 5).

#### 2.3.4. Risk Assessment Code

The mobility and bioavailability of sediment heavy metals can be characterized using the risk assessment coding method. This method was proposed based on the different forms in which heavy metals occur in sediment, as well as the variations in their binding forces. The F1 form, characterized by weak bonding, can easily be exchanged with the overlying water and has high bioavailability [32]. Therefore, this method evaluates F1 in heavy metals by assessing the proportion of the acid extractable form in the total amount of heavy metals. Subsequently, it evaluates the environmental risks associated with them [33,34]. The classification of risk levels is as follows: no risk (<1%), low risk (1–10%), medium risk (11–30%), high risk (31–50%), and extremely high risk (>50%).

#### 2.3.5. Human Health Risk Assessment

The health hazards resulting from ice and water were assessed using the health risk assessment model recommended by the USEPA. In this study, the population in the study area was categorized into adults and children based on age, and the carcinogenic risk and non-carcinogenic risk were evaluated separately. Arsenic was identified as a carcinogenic heavy metal [35], whereas Hg was identified as a non-carcinogenic heavy metal [36]. The health risks to the human body associated with heavy metals primarily occur through drinking, respiration, and contact. Among these, the health risks arising from the drinking route are generally 2 to 3 orders of magnitude higher than those arising from the respiratory and contact routes. The reason for this result had to do with the choice of units to calculate different parameters in the human health model [37]. Therefore, this study considers the health risks from drinking routes in the health evaluation. The formula for assessing the carcinogenic health risks of heavy metals is as follows [38]:

(3) $$ADD=\frac{C\times IR\times EF\times ED}{BW\times AT}$$

(4) $$CR=ADD\times SF_{i}$$

The formula for calculating the non-carcinogenic health risks of heavy metals is as follows [39]:

(5) $$HQ=\frac{ADD}{RfD_{i}}$$

(6) HI=∑HQ

where *CR* represents the risk associated with the consumption of chemical carcinogens from heavy metals through the drinking water route. *ADD* represents the average daily exposure dose per unit weight produced through the drinking water route, measured in mg/kg/d. *SF_i_* denotes the carcinogenic strength coefficient of carcinogenic heavy metal elements, with a specific value of 1.5 (kg/d)/mg for As [40]. *HQ* is the hazard quotient used to assess the risk of chemical non-carcinogens. An *HQ* value greater than 1 indicates a low non-carcinogenic risk to the human body. *RfD* represents the average daily reference dose of chemically non-carcinogenic metal elements through the drinking water route. For Hg, the *RfD* value is 0.0003 mg/kg/d [41]. *C* represents the concentration of heavy metal elements in mg/L. *IR* represents the average daily drinking water volume for humans, with a mean value of 2 L/d for adults [42] and a mean value of 0.7 L/d for children [43]. *EF* denotes the exposure frequency to the heavy metal elements, which is 365 days per year (365 d/a). *ED* represents the continuous exposure time to heavy metals. Typically, the continuous exposure time for carcinogenic heavy metals is 70 years (70 a), whereas it is 30 years (30 a) for non-carcinogenic heavy metals. *BW* refers to the average body weight, with the average weight for adults in Inner Mongolia being 66.1 kg [44] and that for children in Inner Mongolia being 22.9 kg [45]. *AT* represents the average exposure time. For adults and children, the exposure times for carcinogenic heavy metals were 25,550 days [46] and 10,950 days [46], respectively. For children, the exposure time for non-carcinogenic heavy metals was 3650 days [46]. The average life expectancy in the study area was 70 years [45,46].

#### 2.3.6. Monte Carlo Simulation and Uncertainty Analysis

Traditional deterministic models are commonly employed in statistical methods. However, these models often overlook the variability of input variables in calculations, resulting in deviations in the obtained health risk assessment results. To address this issue, this study used Monte Carlo simulation, which employs the most suitable probability distribution to account for the inherent randomness within the input variables. This approach yields more meaningful results [47] and enables the correction of potential human health risks. Moreover, it eliminates the limitations caused by the uncertainty and randomness of sample points, as well as the limited and discrete nature of the environmental system’s complexity. Additionally, it allows for the determination of the corresponding probability of different health risks in different areas and the sensitivity of human health risks to different parameters. The main steps involved in Monte Carlo simulation are as follows:

1. Fit the distribution function types of heavy metal data and rank the system based on goodness of fit statistics. Select the optimal fit probability distribution based on the results.
2. Utilize the risk assessment model, which employs the same deterministic method as the input data, for risk prediction.
3. Construct the mathematical model of the potential ecological risk function based on the reference value and toxicity response coefficient. Incorporate the results of each variable into the model to construct the probability density distribution function for potential ecological risk evaluation.
4. Employ the model to calculate the contribution of heavy metals and obtain the risk distribution. In this study, the Oracle Crystal Ball software (version 11.1.24, Oracle, Redwood Shores, CA, USA) was used to perform 10,000 independent iterations, enhancing the accuracy and stability of the results. Additionally, the Oracle Crystal Ball software (version: 11.1.24) was employed to conduct sensitivity analysis, reflecting the impact of each parameter on the risk results. The higher the sensitivity value, the greater the impact on the ecological risk results [48]. The probability distribution of random variables of the model is shown in Table 1.

### 2.4. Statistical Analysis

Basic data processing and calculations were conducted using Excel 2019. Data correlation analysis was performed using SPSS 25. Graphs were generated using Origin 2022. Additionally, map production was carried out using ArcGIS 10.7. To obtain stable probability risk and sensitivity indicator values, 10,000 Monte Carlo simulations were conducted in Oracle Crystal Ball (Version 11.1.24, Oracle, Redwood Shores, CA, USA).

## 3. Results and Discussion

### 3.1. Hg and As in Ice

#### 3.1.1. Distribution of Hg and As in Ice

The average concentration of Hg in the ice was 8.20 ng/L, while that of As was 2.34 μg/L (see Table 2). The Hg concentration in the ice was significantly higher than that in other regions worldwide [50,51,52,53], exceeding the average Hg content in ice by a factor greater than 10 in certain areas. Similarly, the concentration of As in the ice was elevated compared to that in other regions [54,55]. This indicated that heavy metal pollutants also affect the ice during the frozen period. Furthermore, some heavy metal substances in the water enter the ice during the freezing process, emphasizing the need to address the pollution of Hg and As in the ice.

#### 3.1.2. Vertical Distribution of Hg and As in Ice

**Figure 2:**
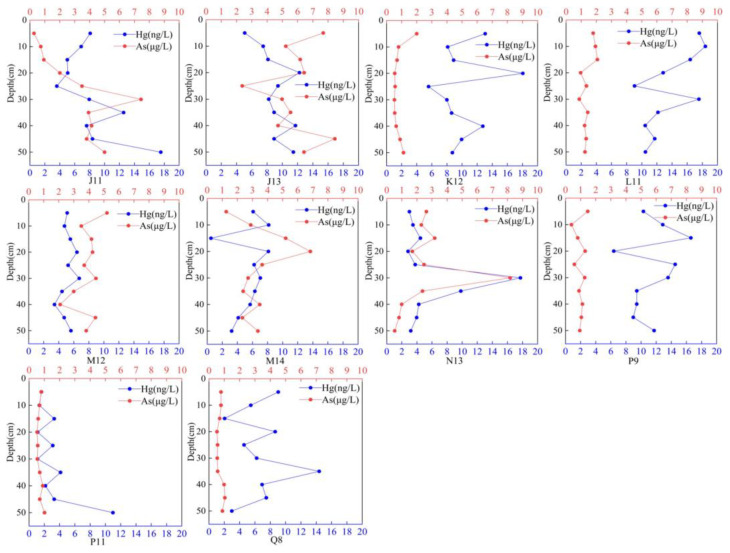
Vertical distribution of Hg and As in ice.

The vertical distribution of the ice revealed that the highest concentration of Hg was found in the 20–40 cm ice layer, specifically at sampling points J13, K12, L11, M14, N13, P9, and Q8. Conversely, the lowest concentration of Hg was observed at the M12 sampling point. The vertical distribution of As in the ice remained relatively stable, with no significant changes at sampling points K12, L11, P9, P11, and Q8. The overall concentration of As at these sampling points was relatively low. The vertical distribution pattern of As at J11, J13, M14, and N13 was similar to that of Hg, with significantly higher concentrations in the middle ice layer compared to the upper and lower layers (see Figure 2).

### 3.2. Hg and As in Water

#### 3.2.1. Concentration Distribution of Hg and As in Water

The concentration of Hg in water of Wuliangsuhai Lake ranged from 0.01 to 0.88 μg/L, with an average concentration of 0.36 μg/L. The concentration of As ranged from 0.48 to 11.3 μg/L, with an average concentration of 5.72 μg/L. The average Hg content in Wuliangsuhai Lake during the frozen period was below the standard for irrigation water quality (GB5084-2021) [29]. It is generally accepted that the Hg content in natural water is typically less than 0.005 μg/L [56,57]. A comparison of Wuliangsuhai Lake with other natural water in China (e.g., lakes, bays, and estuaries (see Table 3)) revealed that the Hg concentration in Wuliangsuhai Lake is relatively high. It is only lower than that in the more severely polluted Chaohu Lake and Dianchi Lake, and higher than nature water such as Dongting Lake.

The concentration of Hg in the Wuliangsuhai Lake is significantly higher than that in the Dajiuhu wetland, Wujiang River, Nanming River, Honghu Lake, Caizi Lake, and surface water in the Shandong Peninsula. Compared with other regions in Asia, the average concentration of Hg in Wuliangsuhai Lake is lower than Iran’s drinking water.

The As content in the water is lower than the standard thresholds for standard for irrigation water quality (GB5084-2021) [29]. Overall, the water is in a clean condition. When compared to natural water in other countries and regions, during the frozen period, the As content in water is lower than that in Taihu Lake and Dianchi Lake. However, it is higher than that in Honghu Lake, Chaohu Lake, and Dongtinghu Lake, among others. Compared with the lakes in the Tibet Plateau, which are located in the same cold region, the As content in the water of Wuliangsuhai Lake is close to fresh water lakes and much lower than that in saline lakes. Compared with other regions in Asia where As pollution is more serious, the arsenic pollution level of Wuliangsuhai Lake is also higher, only lower than Lake Balkhash in Kazakhstan. Therefore, As pollution in Wuliangsuhai Lake also warrants attention.

Overall, the concentration of Hg and As in water during the frozen period is higher than that in lakes worldwide. The average concentration of Hg and As is generally higher than that in natural water less affected by human activities.

#### 3.2.2. Vertical Distribution of Hg and As in Water

**Figure 3:**
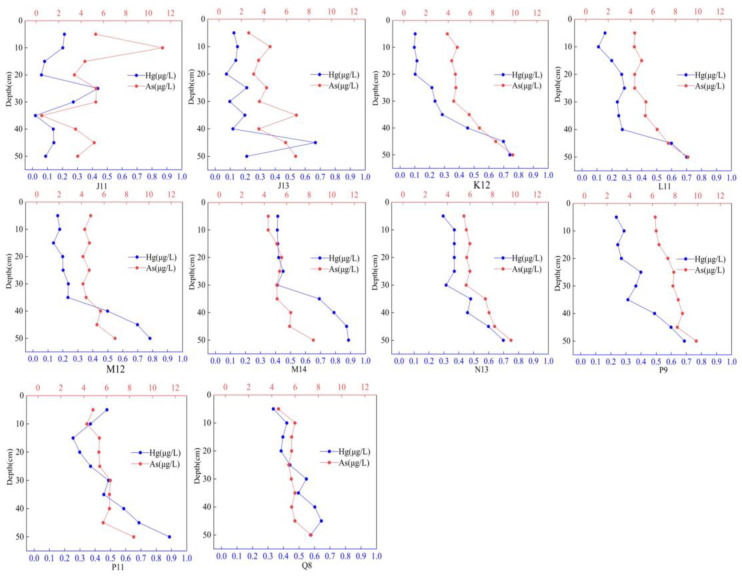
Vertical distribution of Hg and As in water.

The vertical distribution of frozen water is illustrated in Figure 3, which reveals similar distribution patterns for the two substances at the sampling points. With the exception of sampling point J11, the concentrations of Hg and As at all sampling points exhibit a higher concentration at the bottom compared to the surface and middle layers. The concentration generally increases with depth, with a noticeable inflection point between 30 cm and 40 cm. The variation range of Hg concentrations is larger than that of As concentrations. Previous research has indicated that hypoxic conditions can enhance the circulation of As in lake water environments [77]. The presence of ice sheets during the frozen period creates extremely hypoxic conditions in the water, leading to the release of As from the sediment. Moreover, both the groundwater and surface water in the Hetao irrigation area of Wuliangsuhai Lake have a high background value of As [78]. Wuliangsuhai Lake discharges a significant amount of farmland water into the upstream Hetao irrigation area, which may contribute to the high concentration of As in all layers of the water.

### 3.3. Distribution of Hg and As in Sediment

#### 3.3.1. Distribution of Hg and As in Sediments

During the frozen period, the average Hg content in the sediment was 0.035 mg/kg, which is below the TEL. This suggests a low probability of biotoxic effects caused by Hg. Compared to natural water such as lakes, estuaries, and bays in other parts of the world, the Hg content in Wuliangsuhai Lake sediment is relatively low. It is lower than that in typical lakes in China like Taihu Lake, Honghu Lake, and Chaohu Lake, but higher than that in some natural water like Nanming River and Bohai Gulf. In contrast, the As content in glacial sediments is in the range of 3.91–13.00 mg/kg, with an average content of 7.47 mg/kg. The average content of As exceeds the TEL but is lower than the PEL. This suggests that As has the potential to trigger biotoxic effects. Compared to other study areas, the As content during the frozen period is relatively low. It is only higher than that in the Texcoco saline Lakes in Mexico and the Hormozgan Coastal Province in Turkey, but lower than that in Beibu Gulf, and Shandong Peninsula. It is also lower than that in representative lakes like Taihu Lake, Honghu Lake, and Dongting Lake (see Table 4).

#### 3.3.2. Vertical Distribution of Hg and As in Sediments

**Figure 4:**
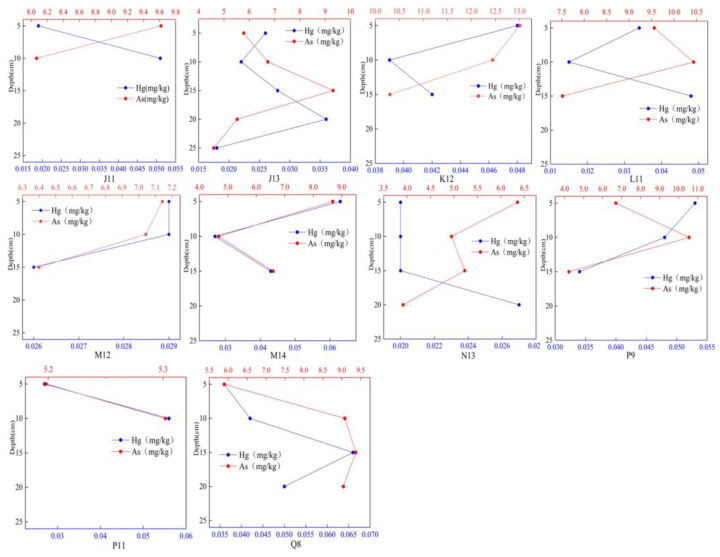
Vertical distribution of Hg and As in sediments.

The vertical distribution of Hg and As in sediments is illustrated in Figure 4. The sediments exhibited a vertical distribution pattern, indicating the depths of J11, P11, N13, and Q8. The Hg content in K12, M12, and P9, as well as that in J13, L11, and M14, displayed significant fluctuations with increasing depth. Conversely, the rate of change in As content was relatively low, observed at sampling points J11, K12, M12, and N13. Additionally, the content at Q8 increased with depth, whereas As was not evident at sampling points J13, L11, M14, P9, and P11.

Overall, there was a greater regularity in the vertical distribution of Hg than in As during the frozen period in sediments. This regularity was observed in the maximum content of both substances at the same depth at the same sampling point. This suggests that similar environmental factors may be influencing the distribution of both substances. Additionally, the vertical distribution patterns of Hg and As in the sediments did not show a clear correlation with the distribution patterns of water and ice, the finding was consistent with the findings of previous studies such as Mekong Delta, Ecuador, and Turky [87,88,89].

#### 3.3.3. Different Fractions and Bioavailability of Hg and As in Sediments

**Figure 5:**
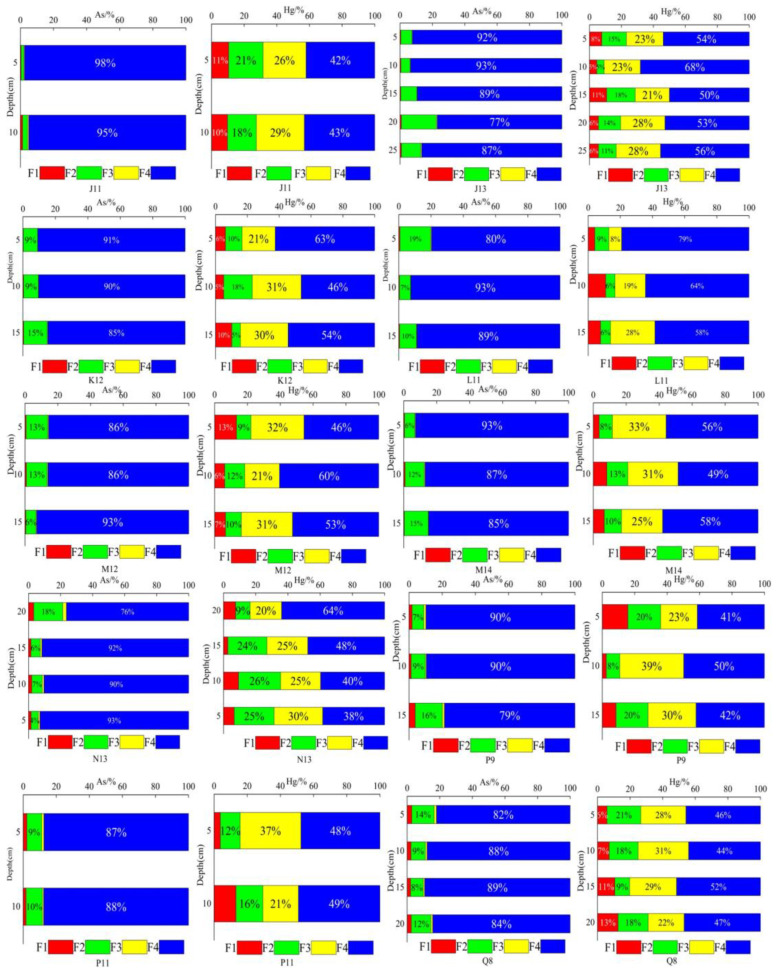
Vertical distribution of different species of Hg and As in sediments.

Figure 5 illustrates the sediments’ fractions distribution of Hg and As. Hg and As were predominantly present in the F4 form in each layer. The average Hg content in the F4 form accounted for 51.6%, with the average proportions of the three convertible forms being 26.8% (F3), 13.4% (F2), and 8.0% (F1). The average As content in the F4 form reached 89.4%, whereas the proportions of the three convertible states were 9.8% (F2), 1.1% (F1), and 0.6% (F3). Comparison of the concentrations of different forms reveals that the proportion of Hg in the non-residue state is higher, indicating its high bioavailability and potential ecological risks to water. Previous studies have demonstrated that organic matter significantly influences the biological activity of Hg [90,91]. During the frozen period, the average organic matter content was 36.9 g/kg, and As existed in the F4 form. The proportion of the F4 form at each sampling point ranged from 76.4% to 97.6%, indicating that As was present in the low-bioavailability form in glacial sediments and that it demonstrated a lower likelihood of causing ecological risks. Among the other forms, the F2 form exhibited the highest proportion. Previous studies have indicated that soil oxides, such as iron, manganese, and aluminum, greatly affect the adsorption of As, which may explain the higher proportion of the F2 form [47,92]. Overall, the distribution pattern of Hg and As during the frozen period was consistent with that observed in previous studies conducted in other regions of China [47,93,94,95].

### 3.4. Pollution Characteristics of Hg and As in Frozen Period

#### 3.4.1. Single-Factor Pollution Index Characteristics

**Figure 6:**
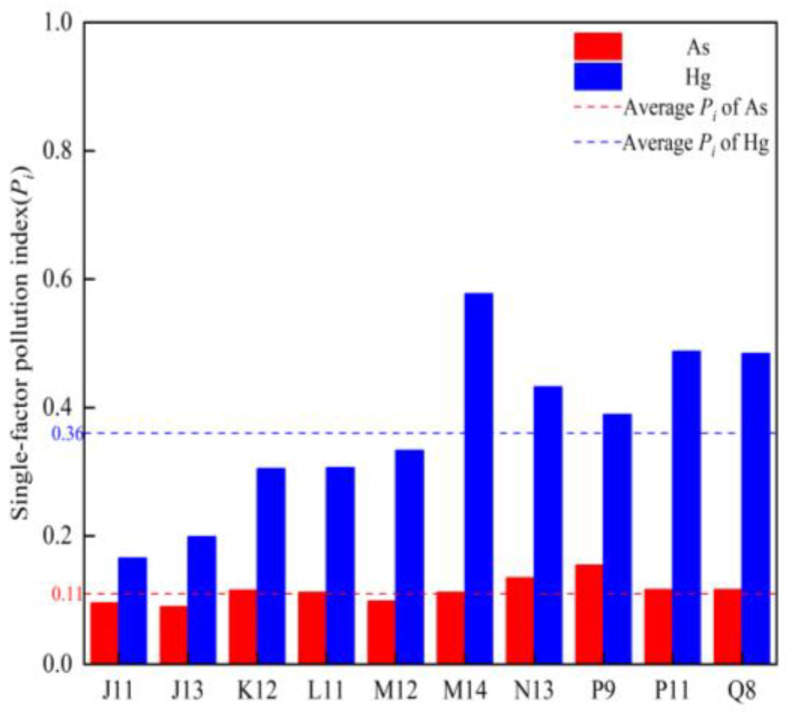
Single-factor pollution index values of Hg and As in the study area.

The single factor index values of both substances were less than 1, indicating the absence of pollution, the average single-factor pollution index of Hg (0.367) was greater than that of As (0.114). In terms of spatial distribution (Figure 6), there was no significant difference in the single factor contamination index between Hg and As at each sampling point (*p* > 0.05). The single factor index values of As at the sampling points followed the sequence of J11 < J13 < K12 < L11 < M12 < P9 < N13 < Q8 < P11 < M14, and those of Hg at the sampling points followed the order of J13 < J11 < M12 < L11 < M14 < K12 < P11 < Q8 < N13 < P9.

#### 3.4.2. Geo-Accumulation Index (*I_geo_*) Characteristics

**Figure 7:**
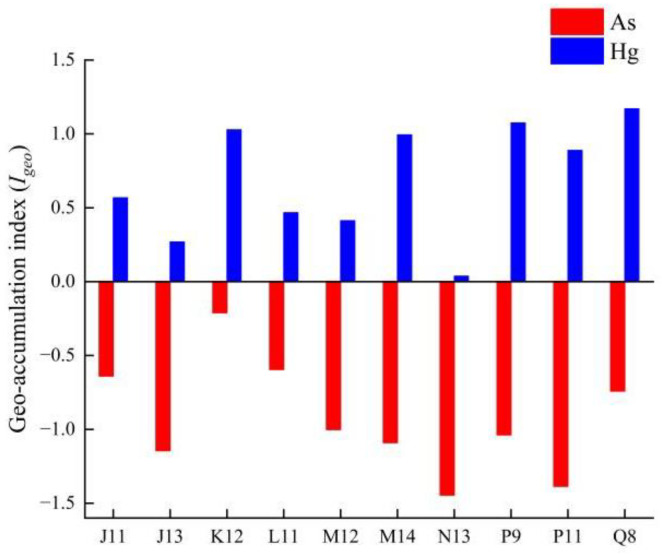
*I_geo_* values of Hg and As in the study area.

The *I_geo_* values of Hg in the sediment of Wuliangsuhai Lake were calculated for 10 sampling sites, the *I_geo_* ranged from −0.485 to 1.652, with an average value of 0.657 (see Figure 7). Overall, the Hg levels indicated that the sediment of Wuliangsuhai Lake was mildly polluted with Hg during the frozen period. At all 10 sampling sites, the *I_geo_* values for As ranged from −1.809 to −0.007, with a mean value of −0.948, indicating that the sediment of Wuliangsuhai Lake was free of As pollution during the frozen period.

#### 3.4.3. Risk Assessment

Table 5 reveals that the bioavailability of Hg in the sediment is high, ranging between 2.4% and 15.8%, with the average value being 7.7%. This indicates that the sediments are classified as low risk. The bioavailability of 25% of the samples exceeds 10%, and they are classified as medium risk, whereas 35% of the samples are classified as low risk. The level of bioutilization of Hg at different sampling points shows that Hg during the frozen period poses certain ecological risks to organisms in the aquatic environment. The bioavailability of As is between 0.2% and 3.7%, with an average value of 1.3%. Compared with Hg, the proportion of As is very low, with 56.3% of the samples being classified as risk free, 43.8% of samples being classified as low risk, and no samples being classified as medium risk or greater. This indicates that As causes little harm to organisms in the lake water environment. Overall, Hg is the main factor for risk of bioutilization in frozen period sediments.

### 3.5. Human Health Risk Assessment

#### 3.5.1. Traditional Health Risk Assessment

Table 6 presents the results of human health risks associated with drinking water. The average carcinogenic risk values for adults and children are 1.06 × 10^−4^ and 1.54 × 10^−4^ in the ice. The non-carcinogenic health risk values for adults have an average value of 7.97 × 10^−4^. The non-carcinogenic health risk values for Hg in the ice have a mean value of 1.15 × 10^−3^. The average carcinogenic risk values for adults and children are 2.60 × 10^−4^ and 3.75 × 10^−4^ in the water. The average non-carcinogenic risk values for adults and children are 3.17 × 10^−2^ and 5.35 × 10^−2^ in the water. Overall, the ecological health risk posed by water is generally higher than that posed by ice. The carcinogenic health risk caused by As exceeds the maximum acceptable threshold of 1.0 × 10^−4^, indicating that As poses a carcinogenic hazard to the population during the frozen period. Additionally, the non-carcinogenic health risk value (HI) caused by Hg is less than 1 in both ice and water, suggesting that Hg does not pose a non-carcinogenic health risk to the population during the frozen period. Therefore, As should be prioritized as the key heavy metal in the environmental health risk management of Wuliangsuhai Lake water during the frozen period.

#### 3.5.2. Probabilistic Health Risk Assessment

**Figure 8:**
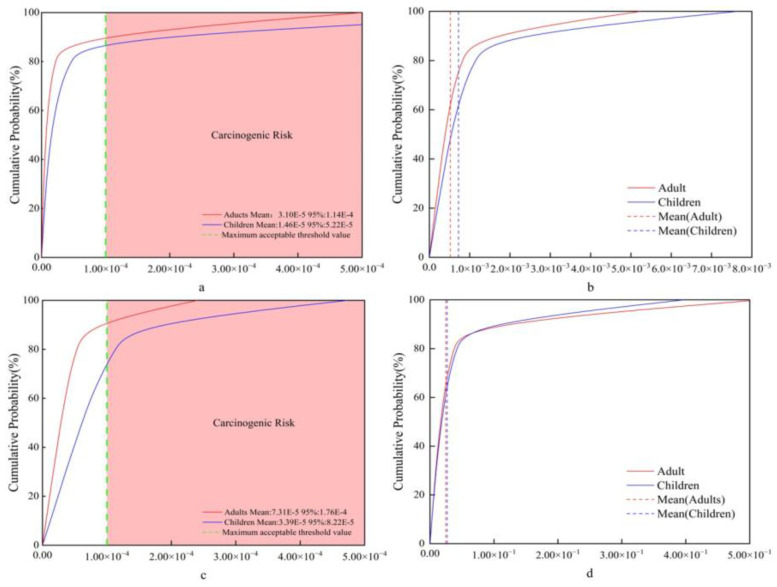
Probability distribution for total carcinogenic risk in (**a**) ice and (**c**) water for children and adults. Probability distribution for total non-carcinogenic risk in (**b**) ice and (**d**) water for children and adults.

Figure 8 illustrates the probabilistic distribution of human health risk obtained after the Monte Carlo simulation. Among the risks of cancer caused by As in ice, the 95% percentile value of carcinogenic health risk in adults exceeds the maximum acceptable threshold set by USEPA (1.0 × 10^−4^), with a value of 1.14 × 10^−4^. The mean and intermediate values are 3.10 × 10^−5^ and 1.50 × 10^−5^, respectively. The 95% centile value of carcinogenic health risk in children from As in ice is 5.22 × 10^−5^, which is below the maximum acceptable threshold set by the USEPA (1.0 × 10^−4^). The mean and intermediate values for children are 1.46 × 10^−5^ and 7.07 × 10^−6^, respectively. This indicates that the risk of As for adults in ice is higher than that for children, possibly due to the lower water intake in children. Furthermore, the non-carcinogenic risk caused by Hg in ice for both adults and children is below 1, suggesting that the health risk posed by Hg to the population is negligible.

In water, the 95% percentile of adult carcinogenic risk is 1.76 × 10^−4^, which is 1.76 times the maximum acceptable carcinogenic risk threshold; 28.2% of adults have a carcinogenic risk higher than the threshold, indicating that As in water poses a certain carcinogenic risk to adults. The 95% percentile of carcinogenic risk for children in water is 8.22 × 10^−5^, and the probability of exceeding 1.0 × 10^−4^ is only 2.5%, indicating that the carcinogenic risk of As in water for children is relatively small. In the non-carcinogenic health risks caused by Hg in water, the probability of health risks for both adults and children is less than 1, indicating that the non-carcinogenic risks caused by Hg in water during the frozen period can be ignored for recipient populations.

#### 3.5.3. Parameter Sensitivity Analysis

**Figure 9:**
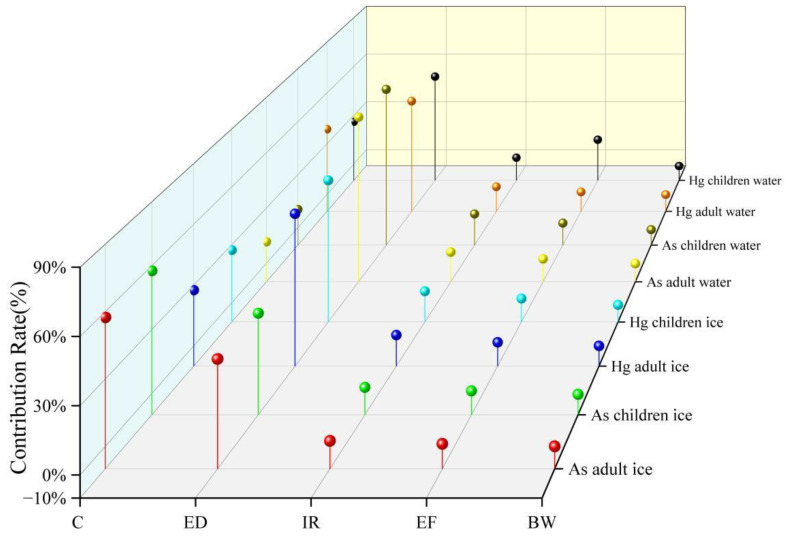
Sensitivity analysis of different exposure factor parameters.

The pollutant content C and exposure time ED in ice and water are the main factors affecting the health risks of recipient populations (see Figure 9). Specifically, the contribution rates of ED to the health risks of cancer in adults and children are 79.3% and 78.2%, indicating that reducing the concentration of drinking As and controlling human exposure to water and ice are two key measures to reduce the health risks of cancer in recipient populations. Among the human non-carcinogenic health risks caused by Hg, the contribution rates of exposure duration ED in ice to adult and child non-carcinogenic health risks are 65.2% and 63.4%, and the contribution rates of exposure duration ED in water to adult and child non-carcinogenic risks are 55.2% and 53.8%. The contribution rate of pollutant content C to human health risks exceeds 10%. This finding aligns with previous research conclusions by Yang et al. [47] and Zhang et al. [96]. Thus, differences are observed in the health risks associated with As and Hg, which may be attributed to the distinct geochemical properties of the freeze–thaw process of these two heavy metals during the frozen period. Additionally, the quantity of water consumed by individuals can impact health risks and should be taken into consideration.

## 4. Conclusions

In this study, the stratified detection and distribution of As and Hg in ice, water, and sediment were used to explore the level and vertical distribution of two toxic substances. The results show that the content of Hg and As in ice is lower than the standard threshold for environmental quality of surface water, and the average content of Hg in water is lower than the threshold for farmland irrigation water quality (GB5084-2021) and higher than the threshold for surface water environmental quality (GB3838-2002). The vertical distribution of As and Hg in ice and water shows similar patterns, and the As and Hg content of the intermediate layer is generally higher than that of the upper and bottom layers. The average content of As in sediments is greater than the limit of TEL in SQGs and lower than the limit of PEL. The content of Hg is lower than the limit of PEL. Furthermore, the content of As and Hg in sediments is lower than that in sediments in other parts of the world. As and Hg show similar patterns in their vertical distribution, and the proportion of non-residual Hg in the sediment is high, with high bioavailability, making it easy to release into water and cause pollution. The ground cumulative index shows that the mean value of *I_geo_* was 0.657, indicating a low risk level, which is consistent with the evaluation results of the ecological risk coding method.

The evaluation of human carcinogenic and non-carcinogenic risks associated with water and ice was conducted using the traditional receptor model and Monte Carlo simulation. The results indicate that the non-carcinogenic risk posed by Hg is low. In the traditional models, the average carcinogenic risk caused by As in ice and water to adults is 1.06 and 2.60 times higher than the maximum acceptable values set by the USEPA, respectively. Similarly, the mean carcinogenic risk caused by As in ice and water to children is 1.54 and 3.75 times higher than the maximum acceptable value. The Monte Carlo calculation shows that the 95% health risk value in the study area is one to two orders of magnitude lower than that estimated by the traditional receptor model. The average carcinogenic risk for both adults and children falls within the acceptable range. Sensitivity analysis reveals that the duration of continuous exposure and the concentration of As and Hg in the water are the most significant factors influencing human health risk.

This study uses two toxic substances, As and Hg, as representative elements to preliminarily explore the distribution characteristics of toxic substances during the ice sealing period of lakes in cold and arid regions and their impact on human health. It not only clarifies the pollution characteristics of lakes during the ice sealing period, but also provides beneficial supplements for ecological protection of lake basins. In the future, efforts should be made to strengthen pollution control of upstream agricultural water, reduce the use of traditional pesticides, enhance upstream water quality, and reduce pollution in Wuliangsuhai Lake. In later work, the number of tests should be increased and the distribution of different heavy metals in the water environment should be analyzed. In addition, samples of animals and plants in the water environment should be collected to assess the ecological risk effects in more depth.

## Figures and Tables

**Table 1:** Exposure factor parameters in risk assessment model.

Parameter	Unit	Distribution Pattern	Distribution		References
			Adult	Children	
AT	d	Point distribution	Carcinogenic: 25,550 Non-carcinogenic: 25,550	Carcinogenic: 25,550 Non-carcinogenic: 3650	[48]
BW	kg	Log-normal distribution	(59.78, 1.07)	(16.68, 1.48)	[41,49]
IR	L/d	Log-normal distribution	(1.23, 0.27)	(1.12, 0.27)	[41,49]
ED	a	Uniform distribution	(0, 70)	(0, 10)	[41,50]
EF	d/a	Triangular distribution	Min: 180 possible value: 350 Max: 365		[41,49]
RfDi	mg/kg × a	Point distribution	0.0003(Hg)		[41,49]
SFi	kg × d/mg	Point distribution	1.5(As)		[41,49]
Hg ice	ng/L	Log-normal distribution	(7.90, 4.35)		This study
As ice	μg/L	Log-normal distribution	(2.48, 3.22)		This study
Hg water	μg/L	Log-normal distribution	(0.37, 0.23)		This study
As water	μg/L	Log-normal distribution	(5.72, 1.70)		This study

**Table 2:** Comparisons of Hg and As concentrations in ice in Wuliangsuhai Lake with those in other regions.

Location	Region and Country	Hg (ng/L)	As (μg/L)	Reference
Wuliangsuhai Lake	China	8.20	2.34	This study
Arctic Ocean	Sweden	0.54	-	[50]
Weddell Sea	Antarctica	4.70	-	[51]
High Arctic (2015)	Canada	3.20	-	[52]
High Arctic (2014)	Canada	3.68	-	[53]
Tupungatito Glacier	Chile	-	0.96	[54]
West Antarctic	Chile	-	4.32	[55]

**Table 3:** Comparison of Hg and As values in water from Wuliangsuhai Lake with those in water from other regions.

Location	Region and Country	Hg (μg/L)	As (μg/L)	Reference
Wuliangsuhai Lake	China	0.36	5.72	This study
Taihu Lake	China	0.03	12.4	[58,59]
Honghu Lake	China	0.01	3.99	[60]
Chaohu Lake	China	0.74	0.33	[61]
Dongting Lake	China	0.04	3.62	[60,62]
Dianchi Lake	China	10.0	15.0	[63]
Caohai Wetland	China	0.07	1.94	[64]
Dajiuhu Wetland	China	0.01	0.53	[65]
Sheyang Estuary	China	0.03	1.83	[66]
Nanming River	China	0.01	1.40	[67]
Wujiang River	China	0.03	0.91	[68]
Bhairab River	Bangladesh	-	4.09	[69]
Iran’s drinking water	Iran	0.7	2.30	[70]
Lake Balkhash	Kazakhstan	-	40.27	[71]
Fresh water lakes in central Tibetan Plateau	China	-	4.90	[72]
Saline lakes in central Tibetan Plateau	China	-	980.56	[72]
Greek surface waters	Greece	0.05	30.0	[73]
Caizi Lake	China	0.04	3.21	[74]
Beibu Gulf	China	0.10	0.74	[75]
Shandong Peninsula	China	0.04	0.98	[76]

- means no relevant data.

**Table 4:** Comparison of Hg and As values in sediments from Wuliangsuhai Lake with those in sediments from other regions.

Location	Region and Country	Hg (mg/kg)	As (mg/kg)	Reference
Wuliangsuhai Lake	China	0.04	7.47	This study
Taihu Lake	China	0.10	13.3	[79]
Honghu Lake	China	0.16	31.7	[60]
Chaohu Lake	China	0.13	12.0	[60]
Dongting Lake	China	0.18	29.2	[80]
Dianchi Lake	China	0.89	30.5	[63]
Caohai Wetland	China	0.24	12.9	[81]
Dajiuhu Wetland	China	0.06	11.6	[65]
Sheyang Estuary	China	0.02	12.9	[66]
Lhasa River	China	0.03	22.4	[82]
Nanming River	China	0.03	12.9	[67]
Wujiang River	China	0.19	15.2	[68]
Ankobra Estuary	Ghana	0.28	50.1	[83]
Erhai Lake	China	0.17	26.9	[84]
Texcoco saline Lakes	Mexico	0.75	0.43	[85]
Hormozgan Province Coastal	Turkey	0.02	5.92	[86]
Caizi Lake	China	0.05	41.0	[74]
Beibu Gulf	China	0.06	7.82	[75]
Shandong Peninsula	China	0.03	7.84	[76]
TEL		0.17	5.90	[26]
PEL		0.48	17.0	[26]

**Table 5:** RAC (Risk Assessment Code) values (%) in Hg and As in sediments.

Sampling Point	Depth (cm)	As	Hg	Sampling Point	Depth (cm)	As	Hg
J11	5	0.5%	10.5%	M14	5	0.5%	3.5%
	10	1.3%	9.8%		10	0.8%	8.2%
	15	-	-		15	0.4%	6.7%
	20	-	-		20	-	-
	25	-	-		25	-	-
J13	5	0.5%	7.7%	N13	5	1.5%	6.6%
	10	0.5%	4.5%		10	2.0%	9.4%
	15	0.5%	10.7%		15	1.5%	2.6%
	20	0.8%	5.6%		20	3.2%	7.5%
	25	0.8%	5.5%		25	-	-
K12	5	0.2%	6.3%	P9	5	1.8%	15.8%
	10	0.4%	5.1%		10	1.3%	2.5%
	15	0.4%	10.3%		15	3.7%	8.4%
	20	-	-		20	-	-
	25	-	-		25	-	-
L11	5	0.6%	4.1%	P11	5	2.2%	3.8%
	10	0.4%	10.2%		10	1.7%	13.1%
	15	0.5%	7.6%		15	-	-
	20	-	-		20	-	-
	25	-	-		25	-	-
M12	5	0.6%	13.1%	Q8	5	2.8%	5.8%
	10	0.8%	6.1%		10	2.3%	7.1%
	15	0.6%	6.6%		15	2.1%	10.6%
	20	-	-		20	2.5%	12.5%
	25	-	-		25	-	-

**Table 6:** Health risk values.

Element	Ice			Water		
	Adult	Children	Mean	Adult	Children	Mean
As	2.19 × 10^−5^~5.12 × 10^−4^	3.16 × 10^−5^~7.39 × 10^−4^	1.06 × 10^−4^, 1.54 × 10^−4^	2.19 × 10^−5^~5.12 × 10^−4^	3.16 × 10^−5^~7.39 × 10^−4^	2.60 × 10^−4^, 3.75 × 10^−4^
Hg	5.04 × 10^−5^~1.86 × 10^−3^	7.82 × 10^−5^~2.68 × 10^−3^	7.97 × 10^−4^, 1.15 × 10^−3^	1.94 × 10^−4^~8.95 × 10^−2^	2.79 × 10^−3^~1.29 × 10^−1^	3.17 × 10^−2^, 5.35 × 10^−2^

## Data Availability

The dataset used in this study is not publicly available due to a data privacy agreement with Inner Mongolia Agriculture University. However, it can be obtained from the corresponding author upon reasonable request.
